# Investigating the association between diet and risk of head and neck cancer in Taiwan

**DOI:** 10.18632/oncotarget.22010

**Published:** 2017-10-24

**Authors:** Chan-Chi Chang, Wei-Ting Lee, Yao-Chou Lee, Cheng-Chih Huang, Chun-Yen Ou, Yu-Hsuan Lin, Jehn-Shyun Huang, Tung-Yiu Wong, Ken-Chung Chen, Jenn-Ren Hsiao, Yu-Cheng Lu, Sen-Tien Tsai, Yu-Hsuan Lai, Yuan-Hua Wu, Wei-Ting Hsueh, Chia-Jui Yen, Shang-Yin Wu, Jang-Yang Chang, Sheen-Yie Fang, Jiunn-Liang Wu, Chen-Lin Lin, Ya-Ling Weng, Han-Chien Yang, Yu-Shan Chen, Jeffrey S. Chang

**Affiliations:** ^1^ Department of Otolaryngology, National Cheng Kung University Hospital, College of Medicine, National Cheng Kung University, Tainan 70456, Taiwan; ^2^ Institute of Clinical Medicine, College of Medicine, National Cheng Kung University, Tainan 70456, Taiwan; ^3^ Division of Plastic and Reconstructive Surgery, Department of Surgery, National Cheng Kung University Hospital, College of Medicine, National Cheng Kung University, Tainan 70456, Taiwan; ^4^ Department of Stomatology, National Cheng Kung University Hospital, College of Medicine, National Cheng Kung University, Tainan 70456, Taiwan; ^5^ Department of Radiation Oncology, National Cheng Kung University Hospital, College of Medicine, National Cheng Kung University, Tainan 70456, Taiwan; ^6^ Division of Hematology/Oncology, Department of Internal Medicine, National Cheng Kung University Hospital, College of Medicine, National Cheng Kung University, Tainan 70456, Taiwan; ^7^ National Institute of Cancer Research, National Health Research Institutes, Tainan 70456, Taiwan; ^8^ Department of Nursing, National Cheng Kung University Hospital, College of Medicine, National Cheng Kung University, Tainan 70456, Taiwan

**Keywords:** head and neck cancer, diet, vegetables and fruits, risk factor, Taiwan

## Abstract

Most studies reporting an inverse association between the consumption of vegetables and fruits and head and neck cancer (HNC) risk were conducted in Western populations and only a few included East Asians. The current case-control study investigated the association between diet and HNC risk using data of 838 HNC cases and 998 controls from a case-control study of HNC conducted in Taiwan. Each participant was asked about their consumption of fresh vegetables, pickled vegetables, fresh fruits, citrus fruits, meat, processed meat, fish, egg, and dairy products. Unconditional logistic regression was performed to estimate the odds ratio (OR) and 95% confidence interval (CI) of HNC risk associated with each food category, adjusted for sex, age, education, and use of alcohol, betel quid and cigarette. An inverse association was observed between HNC risk and daily intake of fresh vegetables (OR = 0.44, 95% CI: 0.20-0.95, *p*-trend = 0.002) or fruits (OR = 0.55, 95% CI: 0.43-0.72, *p*-trend = 0.00001). Individuals who did not consume fresh fruits and vegetables daily had more than double the risk of HNC compared to those with daily intake of vegetables and fruits (OR= 2.24, 95% CI: 1.54-3.25). The results of the current study supported an inverse association between the consumption of fresh vegetables and fruits and HNC risk. In addition to cessation of cigarette smoking and betel quid chewing and reduction of alcohol drinking, a public health campaign for preventing the occurrence of HNC should promote a healthy diet that contains plenty of fresh vegetables and fruits.

## INTRODUCTION

Head and neck cancer (HNC) consists of cancers of the oral cavity, oropharynx, hypopharynx, and larynx and together these cancers make up the fifth leading cancer in the world, with approximately 600,000 incident cases diagnosed worldwide annually [1]. HNC is also one of the leading cancers in Taiwan, particularly among men. In 2014, oral, oropharyngeal, and hypopharyngeal cancers together ranked as the sixth most common cancer in Taiwan and as the fourth most common cancer among Taiwanese men [2]. The risk factors of HNC are well-established and the occurrence of most HNCs can be attributed to the consumption of alcohol, betel quid, and cigarette [3]. In addition, the incidence of human papillomavirus-associated oropharyngeal cancer has been increasing [4]. In contrast to the well-known risk factors of HNC, less is known about the factors associated with a reduced HNC risk. The only factor that has been consistently associated with a reduced HNC risk is the consumption of fruits and vegetables [5]. In a pooled analysis of 14,520 HNC cases and 22,737 controls from studies conducted in North American, South America, Europe, and Asia, the highest quartile of fruit (odds ratio (OR) = 0.52, 95% confidence interval (CI): 0.43-0.62, *p*-trend <0.01) and vegetable (OR = 0.66, 95% CI: 0.49-0.90, *p*-trend = 0.01) consumption was associated with a lower HNC risk compared to the lowest quartile [6].

Most studies published to date on the role of diet in HNC risk focused on Western populations and only a few originated from East Asian countries. Compared with a Western diet, the Asian diet tends to contain more vegetables and fruits and less meat. Although Taiwan has experienced westernization of lifestyle in recent decades, according to the 1993-1996 and 2005-2008 Nutrition and Heath Surveys in Taiwan (NAHSIT), Taiwanese people increased their consumption of vegetables and fruits, soy products, fish, whole grain, nuts and seeds, while decreasing the consumption of red meat, carbohydrates, and sodium-containing foods, and the intake of dairy products has remained low [7]. Given the difference in dietary patterns between Taiwanese and Western populations, it is interesting to consider whether diet plays a similar role in HNC occurring in Taiwan compared to that in Western populations. Furthermore, in addition to alcohol and cigarette, which are the major contributors to HNC in Western countries, betel quid is another major cause of HNC in Taiwan [8]. It is unclear whether the relationship between diet and HNC, particularly the inverse association between vegetables and fruits and HNC, might differ according to the status of betel quid use.

The aim of the current study was to investigate the association between diet and HNC using data from an ongoing hospital-based case-control study of HNC in Taiwan. In addition, we examined whether the relationship between diet and HNC risk might differ according to the use of alcohol, betel quid, and cigarette.

## RESULTS

HNC cases and controls were not significantly different (*p* > 0.05) in mean age and distribution of sex (Table 1). Controls had significantly more years of education compared to the cases (*p* < 0.0001), with 65% of the controls having completed at least a high school education vs. 43% of the cases. More HNC cases were users of alcohol, betel quid, or cigarette compared to the controls (*p* < 0.0001).

**Table 1:** Demographic and lifestyle characteristics of the head and neck cancer patients and control subjects

Characteristics	Case N=838 n (%)	Control N=998 n (%)	*P*
Age (years)			
Mean (SE)	55.5 (0.4)	54.7 (0.3)	0.09
**Sex**			
Men	786 (93.8)	948 (95.0)	0.27
Women	52 (6.2)	50 (5.0)	
**Education**			
≤ Elementary school	228 (27.2)	170 (17.0)	<0.0001
Junior high	250 (29.8)	177 (17.7)	
High school/Technical school	276 (33.0)	359 (36.0)	
Some college or more	84 (10.0)	292 (29.3)	
**Alcohol drinking**			
Never + occasional	273 (32.6)	543 (54.4)	<0.0001
Former regular	116 (13.8)	113 (11.3)	
Current regular	449 (53.6)	342 (34.3)	
			
Never	245 (29.3)	476 (47.7)	<0.0001
1 drink or less per month	28 (3.3)	66 (6.6)	
1-2 drinks per week	37 (4.4)	66 (6.6)	
3-5 drinks per week	48 (5.7)	71 (7.1)	
Daily drinkers	461 (55.0)	312 (31.3)	
Unknown	19 (2.3)	7 (0.7)	
**Betel quid chewing**			
Never	241 (28.8)	713 (71.5)	<0.0001
Former	317 (37.8)	185 (18.5)	
Current	280 (33.4)	99 (9.9)	
Unknown	0 (0.0)	1 (0.1)	
			
Never	241 (28.8)	713 (71.5)	<0.0001
0.1-9.9 pack-years	122 (14.6)	100 (10.0)	
10.0-19.9 pack-years	105 (12.5)	51 (5.1)	
20.0-29.9 pack-years	93 (11.1)	40 (4.0)	
30.0 or more pack-years	256 (30.5)	90 (9.0)	
Unknown	21 (2.5)	4 (0.4)	
Pack-years (SE)	26.6 (1.3)	7.6 (0.7)	<0.0001
**Cigarette smoking**			
Never	114 (13.6)	326 (32.7)	<0.0001
Former	158 (18.9)	205 (20.5)	
Current	565 (67.4)	466 (46.7)	
Unknown	1 (0.1)	1 (0.1)	
			
Never	114 (13.6)	326 (32.7)	<0.0001
0.1-9.9 pack-years	39 (4.6)	78 (7.8)	
10.0-19.9 pack-years	81 (9.7)	133 (13.3)	
20.0-29.9 pack-years	140 (16.7)	128 (12.8)	
30.0 or more pack-years	453 (54.1)	327 (32.8)	
Unknown	11 (1.3)	6 (0.6)	
Pack-years (SE)	35.8 (1.0)	23.0 (0.8)	<0.0001

Abbreviations: N: number, SE: standard error.

Table 2 presents the association between plant-based food and HNC risk. Consumption of fresh vegetables or fresh fruits was associated with a significantly reduced HNC risk with a significant *p*-trend = 0.002 for fresh vegetables and a *p*-trend = 0.00001 for fresh fruits. Consumption of pickled vegetables or citrus fruits was not associated with HNC risk. In the subsite analysis, consumption of fresh vegetables was associated with a reduced risk of oral cancer and pharyngeal cancer but not laryngeal cancer. Consumption of fruits was associated with a reduced risk across all subsites of HNC.

**Table 2:** The association between plant-based food and head and neck cancer

Diet	Control N = 998 n (%)	Head and neck cancer N = 838 n (%)	OR(95% CI)^a^	Oral cancer N = 525 n (%)	OR(95% CI)^a^	Pharyngeal cancer N = 214 n (%)	OR(95% CI)^a^	Laryngeal cancer N = 99 n (%)	OR(95% CI)^a^
**Fresh vegetables**									
≦once/week	11 (1.1)	31 (3.7)	Reference	20 (3.8)	Reference	9 (4.2)	Reference	2 (2.1)	Reference
2-4 times/week	74 (7.4)	124 (14.8)	0.66 (0.29-1.53)	77 (14.7)	0.59 (0.24-1.45)	31 (14.6)	0.53 (0.16-1.77)	16 (16.3)	1.47 (0.26-8.41)
Daily	913 (91.5)	681 (81.5)	0.44 (0.20-0.95)	428 (81.5)	0.38 (0.17-0.87)	173 (81.2)	0.37 (0.12-1.12)	80 (81.6)	0.87 (0.17-4.61)
			P-trend = 0.002		P-trend = 0.002		P-trend = 0.03		P-trend = 0.21
**Pickled vegetables**									
≦once/week	935 (94.0)	789 (94.5)	Reference	500 (95.6)	Reference	198 (92.9)	Reference	91 (91.9)	Reference
2-4 times/week	49 (4.9)	40 (4.8)	0.87 (0.53-1.42)	21 (4.0)	0.79 (0.44-1.43)	14 (6.6)	1.31 (0.65-2.67)	5 (5.1)	0.93 (0.32-2.66)
Daily	11 (1.1)	6 (0.7)	0.57 (0.19-1.77)	1 (0.4)	0.28 (0.05-1.45)	1 (0.5)	0.28 (0.03-2.49)	3 (3.0)	2.00 (0.49-8.20)
			P-trend = 0.29		P-trend = 0.11		P-trend = 0.81		P-trend = 0.51
**Fresh fruits**									
≦once/week	221 (22.2)	348 (41.7)	Reference	213 (40.7)	Reference	92 (43.2)	Reference	43 (43.9)	Reference
2-4 times/week	263 (26.4)	236 (28.3)	0.75 (0.57-0.98)	152 (29.0)	0.79 (0.57-1.08)	58 (27.2)	0.71 (0.46-1.09)	26 (26.5)	0.60 (0.34-1.06)
Daily	513 (51.4)	251 (30.0)	0.55 (0.43-0.72)	159 (30.3)	0.59 (0.43-0.80)	63 (29.6)	0.58 (0.38-0.89)	29 (29.6)	0.39 (0.22-0.69)
			P-trend = 0.00001		P-trend = 0.0006		P-trend = 0.01		P-trend = 0.001
**Citrus fruits**									
≦once/week	893 (89.5)	778 (93.2)	Reference	482 (92.0)	Reference	203 (95.3)	Reference	93 (94.9)	Reference
2-4 times/week	68 (6.8)	43 (5.1)	1.02 (0.65-1.60)	31 (5.9)	1.17 (0.70-1.95)	8 (3.8)	0.68 (0.30-1.55)	4 (4.1)	0.78 (0.26-2.31)
Daily	37 (3.7)	14 (1.7)	0.53 (0.26-1.07)	11 (2.1)	0.70 (0.32-1.54)	2 (0.9)	0.37 (0.08-1.60)	1 (1.0)	0.25 (0.03-2.00)
			P-trend = 0.16		P-trend = 0.71		P-trend = 0.11		P-trend = 0.17

Abbreviations: CI: confidence interval, N: number, OR: odds ratio.

a. OR and 95% CI were calculated using unconditional logistic regression, adjusted for sex, age, education, cigarette smoking (pack-year categories), betel quid chewing (pack-year categories), and alcohol drinking (frequency).

Table 3 presents the association between animal-based food and HNC risk. Overall, no significant association was observed between HNC risk and meat, processed meat, fish, egg, or dairy products. For the subsite analyses, the only significant finding was the significant inverse association between consumption of egg and pharyngeal cancer.

**Table 3:** The association between animal-based food and head and neck cancer

Diet	Control N=998 n (%)	Head and neck cancer N = 838 n (%)	OR(95% CI)^a^	Oral cancer N = 525 n (%)	OR(95% CI)^a^	Pharyngeal cancer N = 214 n (%)	OR|(95% CI)^a^	Laryngeal cancer N = 99 n (%)	OR(95% CI)^a^
**Meat**									
≦once/week	94 (9.4)	74 (8.8)	Reference	46 (8.8)	Reference	18 (8.4)	Reference	10 (10.1)	Reference
2-4 times/week	232 (23.3)	202 (24.2)	0.99 (0.65-1.50)	123 (23.5)	0.90 (0.55-1.45)	53 (24.8)	1.29 (0.65-2.54)	26 (26.3)	0.89 (0.38-2.05)
Daily	672 (67.3)	560 (67.0)	1.02 (0.70-1.51)	354 (67.7)	0.89 (0.57-1.39)	143 (66.8)	1.25 (0.66-2.37)	63 (63.6)	0.96 (0.44-2.09)
			P-trend = 0.83		P-trend = 0.67		P-trend = 0.63		P-trend = 0.96
**Processed meat**									
≦once/week	873 (87.5)	724 (86.8)	Reference	446 (85.3)	Reference	186 (86.9)	Reference	92 (94.8)	Reference
2-4 times/week	102 (10.2)	92 (11.0)	1.02 (0.72-1.44)	65 (12.4)	1.17 (0.80-1.73)	24 (11.2)	0.92 (0.53-1.59)	3 (3.1)	0.33 (0.10-1.09)
Daily	23 (2.3)	18 (2.2)	0.90 (0.44-1.84)	12 (2.3)	0.96 (0.42-2.18)	4 (1.9)	0.82 (0.25-2.73)	2 (2.1)	0.51 (0.09-2.77)
			P-trend = 0.91		P-trend = 0.61		P-trend = 0.67		P-trend = 0.08
**Fish**									
≦once/week	179 (17.9)	153 (18.3)	Reference	91 (17.4)	Reference	48 (22.4)	Reference	14 (14.4)	Reference
2-4 times/week	353 (35.4)	243 (29.1)	0.75 (0.55-1.03)	156 (29.8)	0.81 (0.56-1.17)	63 (29.5)	0.65 (0.40-1.06)	24 (24.8)	0.70 (0.34-1.46)
Daily	466 (46.7)	439 (52.6)	0.94 (0.70-1.27)	277 (52.8)	1.03 (0.73-1.45)	103 (48.1)	0.72 (0.46-1.15)	59 (60.8)	0.83 (0.43-1.61)
			P-trend = 0.84		P-trend = 0.51		P-trend = 0.29		P-trend = 0.82
**Egg**									
≦once/week	254 (25.4)	230 (27.7)	Reference	124 (24.0)	Reference	67 (31.3)	Reference	39 (39.8)	Reference
2-4 times/week	452 (45.3)	386 (46.6)	1.02 (0.78-1.33)	248 (48.0)	1.13 (0.83-1.55)	97 (45.3)	0.76 (0.50-1.15)	41 (41.8)	0.84 (0.49-1.43)
Daily	292 (29.3)	213 (25.7)	0.94 (0.70-1.27)	145 (28.0)	1.16 (0.82-1.65)	50 (23.4)	0.60 (0.37-0.97)	18 (18.4)	0.54 (0.28-1.04)
			P-trend = 0.68		P-trend = 0.41		P-trend = 0.04		P-trend = 0.07
**Dairy products**									
≦once/week	684 (68.6)	614 (73.3)	Reference	376 (71.6)	Reference	164 (76.6)	Reference	74 (74.7)	Reference
2-4 times/week	144 (14.4)	107 (12.8)	1.11 (0.81-1.52)	70 (13.3)	1.19 (0.83-1.71)	25 (11.7)	1.07 (0.63-1.81)	12 (12.1)	1.18 (0.59-2.37)
Daily	170 (17.0)	117 (14.0)	0.91 (0.67-1.23)	79 (15.1)	1.02 (0.72-1.45)	25 (11.7)	0.74 (0.44-1.25)	13 (13.1)	0.76 (0.39-1.49)
			P-trend = 0.71		P-trend = 0.71		P-trend = 0.34		P-trend = 0.54

Abbreviations: CI: confidence interval, N: number, OR: odds ratio.

a. OR and 95% CI were calculated using unconditional logistic regression, adjusted for sex, age, education, cigarette smoking (pack-year categories), betel quid chewing (pack-year categories), and alcohol drinking (frequency).

Compared to individuals with daily intake of fresh vegetables and fruits, those who only had daily consumption of fresh vegetables had a 1.45 times increase in HNC risk (OR = 1.45, 95% CI: 1.14-1.84), whereas those who only had daily intake of fresh fruits did not have a significant increase in HNC risk (OR = 1.12, 95% CI: 0.44-2.84) (Table 4). The highest HNC risk was observed for those without daily intake of both fresh vegetables and fruits (OR = 2.24. 95% CI: 1.54-3.25).

**Table 4:** The association between combination of fresh vegetable and fruit consumption and head and neck cancer

Daily intake of freshvegetables	Daily intake offresh fruits	Case N=838 n (%)	Control N=998 n (%)	OR(95% CI)^a^
Yes	Yes	239 (28.7)	499 (50.1)	Reference
Yes	No	440 (52.8)	414 (41.5)	1.45 (1.14-1.84)
No	Yes	12 (1.4)	14 (1.4)	1.12 (0.44-2.84)
No	No	143 (17.1)	70 (7.0)	2.24 (1.54-3.25)

a. OR and 95% CI were calculated using unconditional logistic regression, adjusted for sex, age, education, cigarette smoking (pack-year categories), betel quid chewing (pack-year categories), and alcohol drinking (frequency).

The association between consumption of fresh vegetables or fruits and HNC risk did not vary significantly (*p*-heterogeneity > 0.05) by the use of alcohol, betel quid, or cigarette (Table 5). Consumption of fresh vegetables or fruits was inversely associated with HNC risk regardless of the use of alcohol, betel quid, and cigarette.

**Table 5:** The association between fresh vegetables and fruits and head and neck cancer by the use of cigarette, alcohol, and betel quid

Diet	Case n (%)	Control n (%)	OR (95% CI)^a^	Case n (%)	Control n (%)	OR (95% CI)^a^
	Never cigarette smoker			Ever cigarette smoker		
**Fresh vegetables**						
≦once per week	3 (2.6)	3 (0.9)	Reference	28 (3.9)	8 (1.2)	Reference
2-4 time per week	14 (12.3)	17 (5.2)	1.27 (0.17-9.66)	109 (15.1)	57 (8.5)	0.58 (0.23-1.46)
Daily	97 (85.1)	306 (93.9)	0.43 (0.07-2.76)	584 (81.0)	606 (90.3)	0.44 (0.19-1.05)
			P-trend = 0.02			P-trend = 0.02
P-heterogeneity = 0.25						
Non-daily	17(14.9)	20 (6.1)	Reference	137 (19.0)	65 (9.7)	Reference
Daily	97 (85.1)	306 (93.9)	0.35 (0.16-0.77)	584 (81.0)	606 (90.3)	0.70 (0.50-0.99)
P-heterogeneity = 0.13						
**Fresh fruits**						
≦once per week	27 (23.7)	32 (9.8)	Reference	320 (44.4)	189 (28.2)	Reference
2-4 time per week	29 (25.4)	78 (23.9)	0.48 (0.22-1.03)	207 (28.8)	185 (27.6)	0.80 (0.60-1.08)
Daily	58 (50.9)	216 (66.3)	0.47 (0.24-0.94)	193 (26.8)	296 (44.2)	0.55 (0.41-0.74)
			P-trend = 0.07			P-trend = 0.00007
P-heterogeneity = 0.50						
Non-daily	56 (49.1)	110 (33.7)	Reference	527 (73.2)	374 (55.8)	Reference
Daily	58 (50.9)	216 (66.3)	0.75 (0.46-1.25)	193 (26.8)	296 (44.2)	0.61 (0.47-0.70)
P-heterogeneity = 0.59						
	**Never betel quid chewer**			**Ever betel quid chewer**		
**Fresh vegetables**						
≦once per week	6 (2.5)	5 (0.7)	Reference	25 (4.2)	6 (2.1)	Reference
2-4 time per week	31 (12.9)	41 (5.7)	0.65 (0.16-2.61)	93 (15.6)	33 (11.6)	0.70 (0.26-1.89)
Daily	204 (84.6)	667 (93.6)	0.30 (0.08-1.09)	477 (80.2)	245 (86.3)	0.53 (0.21-1.33)
			P-trend = 0.0008			P-trend = 0.07
			P-heterogeneity = 0.23			
Non-daily	37(15.4)	46 (6.4)	Reference	118 (19.8)	39 (13.7)	Reference
Daily	204 (84.6)	667 (93.6)	0.43 (0.26-0.70)	477 (80.2)	245 (86.3)	0.71 (0.47-1.06)
			P-heterogeneity = 0.10			
**Fresh fruits**						
≦once per week	66 (27.4)	117 (16.4)	Reference	282 (47.5)	104 (36.6)	Reference
2-4 time per week	67 (27.8)	182 (25.6)	0.76 (0.49-1.18)	169 (28.4)	80 (28.2)	0.78 (0.55-1.12)
Daily	108 (44.8)	413 (58.0)	0.60 (0.40-0.90)	143 (24.1)	100 (35.2)	0.53 (0.37-0.75)
			P-trend = 0.01			P-trend = 0.0005
	**Never cigarette smoker**			**Ever cigarette smoker**		
			P-heterogeneity = 0.86			
Non-daily	133 (55.2)	299 (42.0)	Reference	451 (75.9)	184 (64.8)	Reference
Daily	108 (44.8)	413 (58.0)	0.70 (0.51-0.97)	143 (24.1)	100 (35.2)	0.59 (0.43-0.81)
			P-heterogeneity = 0.47			
	**Non-regular alcohol drinker**			**Regular alcohol drinker**		
**Fresh vegetables**						
≦once per week	6 (2.2)	6 (1.1)	Reference	25 (4.4)	5 (1.1)	Reference
2-4 time per week	32 (11.8)	34 (6.3)	1.28 (0.31-5.26)	92 (16.3)	40 (8.8)	0.45 (0.15-1.37)
Daily	234 (86.0)	503 (92.6)	0.72 (0.19-2.67)	447 (79.3)	410 (90.1)	0.32 (0.11-0.93)
			P-trend = 0.07			P-trend = 0.01
			P-heterogeneity = 0.55			
Non-daily	38 (14.0)	40 (7.4)	Reference	117 (20.7)	45 (9.9)	Reference
Daily	234 (86.0)	503 (92.6)	0.58 (0.34-0.98)	447 (79.3)	410 (90.1)	0.64 (0.42-0.96)
			P-heterogeneity = 0.89			
**Fresh fruits**						
≦once per week	89 (32.7)	95 (17.5)	Reference	259 (46.0)	126 (27.7)	Reference
2-4 time per week	82 (30.2)	136 (25.0)	0.77 (0.49-1.20)	154 (27.4)	127 (28.0)	0.74 (0.52-1.05)
Daily	101 (37.1)	312 (57.5)	0.50 (0.33-0.76)	150 (26.6)	201 (44.3)	0.60 (0.42-0.85)
			P-trend = 0.0009			P-trend = 0.004
			P-heterogeneity = 0.92			
Non-daily	171 (62.9)	231 (42.5)	Reference	413 (73.4)	253 (55.7)	Reference
Daily	101 (37.1)	312 (57.5)	0.58 (0.41-0.81)	150 (26.6)	201 (44.3)	0.69 (0.51-0.94)
			P-heterogeneity = 0.92			

a. OR and 95% CI were calculated using unconditional logistic regression, adjusted for sex, age, education, cigarette smoking (pack-year categories), betel quid chewing (pack-year categories), and alcohol drinking (frequency).

## DISCUSSION

In the current analysis, we observed a significant inverse association between HNC risk and consumption of fresh vegetables or fresh fruits. The inverse association was observed across all subsites of HNC except for the null association between fresh vegetables and laryngeal cancer. The highest risk of HNC was observed among individuals who lacked daily intake of both vegetables and fruits. The inverse association between consumption of fresh vegetables and fruits did not differ according to the use of alcohol, betel quid, or cigarette.

Consistent with results from published studies [6], the current study showed that consumption of fresh vegetables and fruits was also associated with a reduced HNC risk among Taiwanese. In a pooled analysis (14,520 HNC cases and 22,737 controls) by Chuang et al. with populations mostly from Western countries, consumption of vegetables (OR = 0.66 for highest vs. lowest quartile, 95% CI: 0.49-0.90, *p*-trend = 0.01) and fruits (OR = 0.52 for highest vs. lowest quartile, 95% CI: 0.43-0.62, *p*-trend < 0.01) was associated with a reduced HNC risk [6]. Bravi et al. reviewed 24 case-controls studies (20 studies from North and South America, Europe and Australia, 3 studies from Iran, and 1 study from Indonesia) of upper aerodigestive tract (UADT) cancer (HNC + esophageal cancer) and found that the most consistent finding was a reduced UADT cancer risk associated with a diet rich in fruits and vegetables or nutrients contained in fruits and vegetables [5]. A large prospective cohort study with 490,802 participants from the United States by Freedman et al. reported an inverse association between total vegetable and fruit intake and HNC (hazard ratio for per serving/day/1000 calories = 0.94, 95% CI: 0.89-0.99) [9]. More recently, a case-control study conducted in a Chinese population reported that more frequent consumption of fruits and vegetables was associated with a reduced HNC risk [10]. Another recent case-control study from China reported a reduced oral cancer risk associated with frequent consumption of leafy vegetables, other vegetables, and fruits [11]. Our results together with evidence from published studies showed that vegetables and fruits are associated with a reduced HNC risk across all ethnicities.

Our results showed that the highest HNC risk occurred among individuals who had neither daily intake of vegetables nor daily intake of fruits. For those who had only daily intake of vegetables but no daily intake of fruits, their risk of HNC was lower than those who had neither daily intake of vegetables nor daily intake of fruits (OR = 0.65, 95% CI: 0.45-0.92), but was higher than those who had both daily intake of fruits and vegetables (OR = 1.45, 95% CI: 1.14-1.84). This suggests that daily intake of fruits and daily intake of vegetables are both necessary to achieve the maximum benefit to reduce the risk of HNC.

Our analyses showed that the inverse association between consumption of fresh fruits and vegetables and HNC risk was not significantly different by the use of alcohol, betel quid, or cigarette. The pooled analysis by Chuang et al. also reported that the reduced risk of HNC associated with consumption of vegetables and fruits was not affected by the status of tobacco use or the intensity of alcohol consumption [6]. In a case-control study, Butler et al. showed that the intake of leafy vegetables was associated with a reduced HNC risk only among non-smokers but not among smokers, whereas consumption of fruits was associated with a reduced HNC risk regardless of the smoking status [10]. In a large cohort study from the United States, Freedman et al. reported that the inverse association between total fruit and vegetable intake was similar by the status of smoking and alcohol use [9]. Overall, regardless of the use of alcohol, betel quid, or cigarette, consumption of fruits and vegetables appeared to remain inversely associated the HNC risk.

In our analysis, we found a significant association between consumption of egg and pharyngeal cancer. Due to the smaller number in the analysis stratified by HNC subsite, we could not rule out chance for this result. The inverse association between HNC and egg has been reported by other studies. Butler et al. reported a reduced HNC risk associated with egg consumption, particularly among non-smokers [10]. Chen et al. reported that eating eggs less than 5 times per week was associated with an increased oral cancer risk (OR = 1.44, 95% CI: 1.21-1.71) [11]. In contrast, Chuang et al. reported that egg intake was associated with an increased HNC risk (highest quartile vs. lowest quartile: OR = 1.48, 95% CI: 1.20-1.82) [6]. Given the inconsistent results on the association between egg intake and HNC risk across studies, additional investigations are needed to clarify this association.

Processed meat, which is classified by the International Agency for Research on Cancer as “carcinogenic to humans” [12], has been associated with an increased HNC risk [6, 10]. However, our result did not show a significant association between processed meat and HNC. The major reason for the null association in our study is likely due to the infrequent consumption of processed meat in our study population. Approximately 87% of our study participants reported eating processed meat once or less per week and only 2% reported daily consumption of processed meat.

This study has several limitations. First, the study participants were asked to recall their dietary habits in the year prior to the diagnosis of HNC or before the interview date (for controls). This might have introduced reverse-causality if the development of HNC had forced HNC patients to change their dietary habits. However, this short time-frame for recalling dietary habits had the advantage of reducing recall errors. Second, recall bias could be an issue in a case-control study because case subjects are often likely to ruminate more heavily on their past exposures that might cause their disease. As a result, recall bias could have biased our results away from the null and overestimated the association between diet (particularly consumption of vegetables and fruits) and HNC. However, because of public health campaigns, Taiwanese are more aware of the association between HNC and use of alcohol, betel quid, and cigarette, and less aware of the role of diet in the development of HNC. Therefore, recall bias in the association between diet and HNC is less likely in our study. Another limitation is that we asked about diet in broad categories instead of using a food frequency questionnaire with individual food items. The diet questions that we asked are a component of a comprehensive questionnaire that also surveys other exposure factors. To keep the interview at a reasonable length to minimize disruption to patients’ hospital visits, we could only ask a limited number of questions on diet. Finally, information bias is another limitation because the dietary questionnaire we used was not validated. Consequently, the risk estimates generated by our study might be imprecise and might only reflect a general trend for the association between diet and HNC risk.

The major strength of the current study is that it provides important data for the role of diet in the risk of HNC in an East Asian population. The majority of the studies published to date on the association between diet and HNC were conducted in Western populations. Given the difference in dietary patterns, it is important to investigate the relationship between diet and HNC in different ethnic populations. Our results provide additional evidence to suggest that consumption of vegetables and fruits is beneficial in reducing HNC risk not only for Western populations but also for Taiwanese. Another strength of the current study is that we collected detailed data on the use of alcohol, betel quid, and cigarette, which are the three major risk factors of HNC. With this information, we were able to show that even after adjusting for alcohol, betel quid, and cigarette, consumption of fresh vegetables and fruits was still significantly associated with a reduced HNC risk. In addition, we were able to show that the inverse association between intake of fresh vegetables and fruits and HNC did not differ according to the status of alcohol, betel quid, or cigarette use. This suggests that consumption of vegetables and fruits might be beneficial in reducing HNC risk even for users of alcohol, betel quid, and cigarette.

In conclusion, the results of the current study supported an inverse association between consumption of fresh vegetables and fruits and HNC risk. In addition to cessation of cigarette smoking and betel quid chewing and reduction of alcohol drinking, a public health campaign for preventing the occurrence of HNC should also promote a healthy diet that contains plenty of fresh vegetables and fruits.

## MATERIALS AND METHODS

The current study received an approval from the institutional review boards of the National Health Research Institutes and the National Cheng Kung University Hospital. Signed informed consent was obtained from each study participant.

### Recruitment of study subjects

The current analysis included data from an ongoing HNC case-control study that started subject recruitment on September 1, 2010 in the Department of Otolaryngology and the Department of Stomatology at the National Cheng Kung University Hospital. Eligible cases included individuals who were diagnosed with pathologically confirmed squamous cell carcinoma of the head and neck, including cancers of the oral cavity, oropharynx, hypopharynx, and larynx (ICD-10 codes: C00-C10, C12-C14, C32), had no history of any cancer diagnosis, were aged 20 years or older, and were able to provide informed consent. To compare the experience of risk factor exposure, controls frequency-matched to cases on sex- and age (±5 years) were recruited. Eligible controls were those who received surgery for non-cancerous conditions that are not associated with the use of alcohol, betel quid and cigarette, had no history of cancer diagnosis, and were aged 20 years or older.

### Data collection by interview

A trained interviewer interviewed each study participant using a standardized questionnaire to collect information on dietary habits in the past year before the diagnosis of HNC for the case subjects or before the interview date for the control subjects. Each subject was asked about the frequency of consumption (≦ once/week, 2-4 times/week, or daily) for four plant-based food categories (fresh vegetables, pickled vegetables, fresh fruits, and citrus fruits) and five animal-based food categories (meat, processed meat, fish, egg, and dairy products). Data on potential confounders, including consumption of alcohol, betel quid, and cigarette, were also collected.

### Statistical analysis

To compare the distributions of demographic characteristics (age, sex, and education) and lifestyle factors (use of alcohol, betel quid, and cigarette) between cases and controls, T-tests (for continuous variables) and chi-squared tests (for categorical variables) were performed.

The association between each food category and HNC risk was evaluated by an OR and 95% CI calculated using unconditional logistic regression, adjusted for sex, age, educational level, alcohol drinking (frequency), betel quid chewing (pack-years), and cigarette smoking (pack-years). The pack-year of cigarette smoking = (number of cigarettes smoked per day/20) x total number of year of cigarette smoking. The pack-year of betel quid chewing = (number of betel quids chewed per day/20) x total number of year of betel quid chewing.

Additional analyses were performed with consumption of fresh vegetables and fruits, which were the food categories that showed a significant inverse association with HNC risk in our analysis. We examined the joint effect of fresh vegetables and fruits by examining the risk of HNC associated with different combinations of fresh vegetable and fruit consumption (yes to daily intake of vegetables/yes to daily intake of fruits; yes to daily intake of vegetables/no to daily intake of fruits; no to daily intake of vegetables/yes to daily intake of fruits; no to daily intake of vegetables/no to daily intake of fruits). Further analyses were conducted to examine the association between consumption of fresh fruits and vegetables stratified by the use of alcohol, betel quid, or cigarette to determine whether the relationship between consumption of fresh fruits and vegetables and HNC risk might differ according to the use of alcohol, betel quid, and cigarette. Heterogeneity between strata of alcohol, betel quid, or cigarette use was evaluated with log-likelihood ratio test comparing the unconditional logistic regression model with the product term (vegetables or fruits x alcohol, vegetables or fruits x betel quid, or vegetables or fruits x cigarette) to the model without the product term.

## Abbreviations

CI: confidence interval
HNC: head and neck cancer
NAHSIT: Nutrition and Heath Surveys in Taiwan
OR: odds ratio
UADT: upper aerodigestive tract
